# Is cultural activity at work related to mental health in employees?

**DOI:** 10.1007/s00420-012-0762-8

**Published:** 2012-03-29

**Authors:** Töres Theorell, Walter Osika, Constanze Leineweber, Linda L. Magnusson Hanson, Eva Bojner Horwitz, Hugo Westerlund

**Affiliations:** 1 Stress Research Institute, Stockholm University, Fack 710632, R 017, SE-106 54 Stockholm, Sweden; 2 Department of Public Health Sciences, Karolinska Institute, Stockholm, Sweden; 3 Department of Public Health, Uppsala University, Uppsala, Sweden

**Keywords:** Emotional exhaustion, Depression, Self-rated health, Work stress, Cultural activity

## Abstract

**Objective:**

To examine relationships between work-based cultural activities and mental employee health in working Swedes.

**Hypothesis:**

A positive relationship between frequent cultural activity at work and good employee health was expected.

**Research design:**

Random sample of working Swedish men and women in three waves, 2006, 2008 and 2010, on average 60 % participation rate.

**Methods:**

A postal questionnaire with questions about cultural activities organised for employees and about emotional exhaustion (Maslach) and depressive symptoms (short form of SCL). Employee assessments of “non-listening manager” and work environment (“psychological demands” and “decision latitude”) as well as socioeconomic variables were covariates. Cross-sectional analyses for each study year as well as prospective analyses for 2006–2008 and 2008–2010 were performed.

**Main outcome and results:**

Lower frequency of cultural activities at work during the period of high unemployment. The effects of relationships with emotional exhaustion were more significant than those with depressive symptoms. The associations were attenuated when adjustments were made for manager function (does your manager listen?) and demand/control. Associations were more pronounced during the period with low unemployment and high cultural activity at work (2008). In a prospective analysis, cultural activity at work in 2008 had an independent statistically significant “protective” effect on emotional exhaustion in 2010. No corresponding such association was found between 2006 and 2008.

**Conclusions:**

Cultural activities at work vary according to business cycle and have a statistical association with mental employee health, particularly with emotional exhaustion.

**Implications for future research:**

There are particularly pronounced statistical protective effects of frequent cultural activity at work on likelihood of emotional exhaustion among employees.

## Introduction

Although there is growing evidence that cultural activities in general may promote health (Cuypers et al. 2011; Cox et al. 2010; Clift et al. 2009; Bygren et al. 1996) there are many unanswered questions regarding possibly beneficial health effects of cultural activities organised through work. In a random trial Bygren et al. (2009a) have shown that an offer of a cultural activity (self-selected from a list of possible activities) once a week for medical staff lasting for 2 months may have beneficial effects on mental health during this period. However, the kinds of cultural activities offered and the way in which such activities are organised may be crucial for the effects. In a study by our group (Theorell et al. 2009) it was shown that among employees who were offered cultural activities once a week for 3 months, those who were the most enthusiastic participants were likely to benefit the most with regard to health but also that social climate (social support) may have been disturbed for these people (a jealousy effect among non-participants?). The conclusion was that cultural activities at work should preferably be organised in such a way that all employees are offered participation and that the majority of employees should be able to benefit. Therefore, it is not known whether cultural activities organised through work are beneficial for employee health or not. The present study was performed in order to throw light on this question.

That regular cultural activities in managers could have important effects on employee health has been shown in a recently published randomised intervention study from our group (Romanowska et al. 2011). A year-long art-based manager education programme was compared with an accepted educational programme designed for improvement of psychosocial competence in managers. The managers themselves as well as their employees were followed from start during the process up to 18 months after start (and half a year after the end of the respective programmes). The results showed that the art-based programme for the managers had more beneficial effects on employee health than the alternative after 18 months, both on standard scores for psychological health and on a the blood concentration of a regenerative hormone (DHEA-s). This shows that arts may have the power to improve managers’ ability to improve their employees’ health.

What are the possible mechanisms behind a relationship between cultural activities organised at work and employee health? This has not been discussed extensively in the scientific literature, but possible health promotion effects of cultural activities in general have been discussed scientifically. Cultural activities may promote creativity (Wikström 1994) and increase cohesiveness in groups (Cuypers et al. 2011). For specific activities, for instance choir singing, there are studies which have shown beneficial psychological and biological effects of choir rehearsals (Sandgren and Borg 2009; Kreutz et al. 2004) as well as of singing lessons (Grape et al. 2003). Similarly, amateur tango dancing stimulates beneficial endocrinological reactions (Quiroga Murcia et al. 2009). More long-lasting endocrinological effects favouring regenerative function have also been shown when the choir participation continues once a week for several months (Grape et al. 2010). In samples of elderly people, there is extensive research showing that choir singing stimulates a feeling that life is worth living and that this motivates participants to assume health promoting life habits (Clift and Hancox 2001; Cohen 2009). All of these possible mechanisms could be relevant for possible effects of cultural activities at work. The workplace, however, is an arena on which cultural activities offered to the employees could have unexpected creative stimulating cultural experiences. Such activities may be different from the ones the employees would choose with family and friends and the context is a different one. Interviews from our own pilot study (Theorell et al. 2009) illustrated that the introduction of a weekly cultural programme for employees “opened eyes” to unexpected worlds for some employees.

In summary, possible health effects of cultural activities in the workplace could arise (1) because such activities may strengthen cohesiveness between employees and between management and employees resulting in improved psychosocial work environment or (2) because of direct effects of the cultural activities themselves.

The present study was designed to illuminate firstly whether cultural activities at work are related to mental health in employees and secondly to what extent possible associations between cultural activities at work and employee health could be explained statistically by indirect effects on psychosocial work environment variables as they are perceived by the employees themselves (“a listening/non-listening manager” and psychological demands and decision latitude). The former type of manager variable has been established in our previous studies as an important explanatory factor in “ongoing conflicts” (Oxenstierna et al. 2011). Psychological demands and decision latitude are well-established variables in the study of work environment variables of relevance to employee health (Karasek and Theorell 1990).

Although the studies by Bygren et al. (1996) indicate that regularly repeated cultural activities during long periods of life are associated with reduced mortality (even after adjustment for a number of possible confounding factors), the duration of such possible effects are largely unknown, particularly in relation to activities organised at work. An additional aim of the present work is therefore to examine whether cultural activities at work may be predictive of improved health also in the near future (2 years, respectively). Finally, the question was raised whether cultural activity at work may be related to business cycle as it is mirrored in unemployment rates in the Swedish society. If so, does this have any consequence for the relationship between cultural activity at work and employee health?

## Study sample and methods

The SLOSH (Swedish Longitudinal Occupational Survey of Health) participants were originally recruited from the Swedish Work Environment Survey (SWES) which is conducted biennially by Statistics Sweden (SCB) and consists of subsamples of gainfully employed people, aged 16–64 years, from the Labor Force Survey (LFS). These individuals were first sampled into the LFS through stratification by county of birth, sex, citizenship, and inferred employment status. The respondents to SWES 2003 and 2005 were invited to enroll in the SLOSH (Kinsten et al. 2007), which was initiated by the Stress Research Institute in 2006. The total response rate in this first wave which included only the SWES respondents in 2003 was 65 %. The second data collection which included both the SWES 2003 and the SWES 2005 respondents was conducted in April 2008 by Statistics Sweden, on behalf of the Stress Research Institute at Stockholm University. A total of 18,734 individuals were mailed self-completion questionnaires in 2008, out of whom 9,756 (52 %) individuals responded. The total response rate of the study was however 11,441 (61 %), including non-working participants (not analysed in the present study). In 2010 the total response rate was 57 %. More detailed information about the cohort, response rate and characteristics of responders versus non-responders has been published elsewhere (Hanson et al. 2008; Nyberg et al. 2008; Kinsten et al. 2007; Hasson et al. 2011). In the samples studied in the present report the average response rate (among working subjects) was 60 %. There was no difference between responders and non-responders with regard to county of birth and citizenship. Numbers of participants as well as age and gender distributions are presented in Table 1. Data collection took place in April–May in all the three waves.

**Table 1:** Characteristics of the study populations

	2006	2008	2010
% women	55	56	56
Age	47.6 (11.6)	49.2 (11.6)	51.6 (11.5)
Ln (income)	5.49 (0.55)	5.59 (0.51)	5.68 (0.54)
Non-listening manager	2.16 (0.77)	2.15 (0.75)	2.20 (0.83)
Demands	11.75 (2.70)	11.62 (2.61)	11.95 (2.67)
Decision latitude	11.56 (2.89)	11.40 (2.72)	11.39 (2.75)
Cultural activity/work	1.52 (0.61)	1.61 (0.65)	1.52 (0.64)
Emotional exhaustion	11.63 (5.93)	11.98 (6.02)	10.76 (5.74)
Depressive symptoms	11.78 (5.30)	11.59 (5.26)	11.78 (5.30)
Number of participants	4,950–5,985	8,801–11,121	8,315–11,525

Means and standard deviations (within parentheses). The minimum number corresponds for all three study years to the number of participants who answered the question about “non-listening manager” since self employed subjects could not answer this question. The maximum number for all three study years corresponds to the number of men and women who only answered small parts of the questionnaire

The following question was used for the assessment of cultural activities at work:

*Are cultural activities (movies, theatre performances, concerts, exhibitions) organised for the employees in your work place?* with response alternatives: 0 = never, 1 = sometimes per year, 2 = sometimes per month, 3 = sometimes per week or more often).

The following explanatory variables were used:

*Age, gender and annual income according to the tax registry* (e log transformed in order for us to obtain close to normal distributions) were included as adjustment variables in all equations. Education had no additional statistical effect and was therefore not included.

The listening/non-listening manager variable was based upon the following question: “*Does your boss listen to you taking in what you are saying?”* with response alternatives 1 = to a very high degree, 2 = to a high degree, 3 = to a small degree and 4 = to a very small degree or not at all.

*Psychological demands and decision latitude* were assessed by means of the Swedish abbreviated version (DCQ) of the demand–decision latitude questionnaire originally introduced by Karasek (Karasek 1979; Theorell et al. 1988; Theorell 1996). There were five questions related to demands (for instance: Does your work require you to work very hard? Do you have enough time to complete your work?) and six questions related to decision latitude (for instance: Are you free to decide what to do at work? Do you get to learn new things at work?). There were four response alternatives for each question ranging from never to always or almost always. Sum score ranges were 5–20 and 6–24, respectively. These are well-established scores. Psychometric properties have been reported by Theorell (1996), with Cronbach alpha >0.70 for both dimensions in the general Swedish working population.

## Health outcome variables

Emotional exhaustion was measured by the Maslach Burnout Inventory, General Survey (MBI-GS), (Leiter and Maslach 1999) using the emotional exhaustion subscale. The scale consists of five items (“Emotionally drained”, “totally exhausted at the end of the working day”, “tired when I get up in the morning to meet a new day”, “really tiring to work a full day”, “burnt out by work”) derived from the Maslach Burnout Inventory human services survey (MBI-HSS) in unmodified form. Response options cover six steps from ‘Every day’ to ‘A few times a year or less/Never’. A sum score ranging from 5 to 30 was calculated—a high score indicates a high level of exhaustion; standard deviation 5.9. Psychometric properties which were shown to be excellent were published for the Swedish version in Hanson et al. (2008).

Depressive symptoms were measured with a brief subscale from the Hopkins Symptom Checklist (SCL-90). The scale measures one-week prevalence and includes six items covering the depressive core symptoms ‘feeling blue’; ‘feeling no interest in things’; ‘feeling lethargy or low in energy’; ‘worrying too much about things’; ‘blaming yourself for things’; ‘feeling everything is an effort’. Response options cover five steps from ‘not at all’ to ‘a great deal’. A sum score of depressive symptoms ranging from 0 to 24 was calculated with a high score indicating a high probability of clinical depression; standard deviation 5.1. Scale characteristics for this short Swedish version which were shown to be excellent have been published by Magnusson Hanson et al. (2009).

## Statistical methods

In the first step product moment correlations were calculated between all explanatory study variables using accumulated scores for both cultural activity and the work-related variables.

Since the study variables were normally or close to normally distributed, multiple linear regressions were performed in the next step using cultural activity as explanatory and health variables as outcome. Each wave was analysed separately. The regressions were made in three successive versions:

1. with adjustment for age, gender and nlog (income) from the actual study year and education from self-reported data in 2006.
2. In addition to 1. also with adjustment for listening/non-listening manager.
3. In addition to 2. also with adjustment for psychological demands and decision latitude at work.

Since income has had a more important role as a confounding factor in the association between cultural activities and health in previous research (Bygren et al. 1996) and since decision latitude and education are strongly correlated, we decided to use income and decision latitude but not education as a proxy for socioeconomic status in the multiple regressions.

A final step was two series of prospective analyses, one from 2006 to 2008 and one from 2008 to 2010, in the form of multiple linear regressions. In these analyses age, gender and logarithmically transformed income as well as the work-related variables listening/non-listening manager, psychological demands and decision latitude and finally cultural activities at work and psychological state (emotional exhaustion and depressive symptoms, respectively) at start of follow-up (2006 and 2008, respectively) were used as predictors and subsequent psychological states (emotional exhaustion and depressive feeling 2008 and 2010, respectively) as outcomes.

## Results

**Fig. 1:**
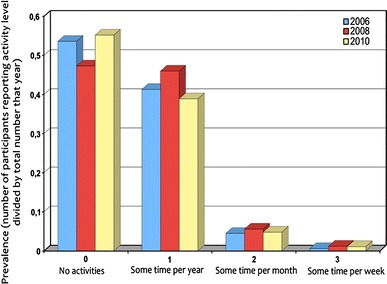
Prevalence of different frequencies of cultural activities at work reported during the three study years. *0* No activities, *1* some time per year, *2* some time per month, *3* some time per week or more often. Swedish Longitudinal Occupational Study of Health, 2006 *n* = 5,037, 2008 *n* = 9,623, 2010 *n* = 8,912

Figure 1 shows the distributions of “cultural activities” for each year as reported by all available participants, disregarding previous participation and information about other variables. About half of the subjects reported that no cultural activities at all had been organised during the year preceding the survey. Among those who reported cultural activities, the most frequent alternative was “sometimes per year”. More frequent cultural activities were accordingly not so frequent: 0.6, 1.2 and 1.1 % in 2006, 2008 and 2010, respectively. There was a significant difference between the study years (ANOVA for repeated measures *F* = 39.34, *df* = 2/2567, *p* < 0.0001). Any cultural activity during the past years was reported by 46.4, 52.7 and 44.8 %, respectively. Accordingly, cultural activities organised through work were the most frequent during the year with the lowest unemployment rate (6 % unemployed nationally both in 2006 and 2008) and the least frequent during the year with the highest unemployment (8.5 % unemployed nationally in 2010 during the spring period when data was collected).

Table 2 shows product moment correlations between all the explanatory and outcome variables. These calculations have been based upon subjects with data from all waves and accumulated scores have been created which means that cultural activity score, exhaustion score, depressive symptom score, psychological demands score and decision latitude score have been summed across the study years and the respective sums used in the calculations of correlations. Age, gender, income and education have been assumed to be constant and are therefore based upon 2006 data. The table shows relatively modest correlations between education, income, non-listening boss and work decision latitude on one hand and cultural activities at work on the other hand, the highest correlation (cultural activity and decision latitude at work) being 0.22.

**Table 2:** Product moment correlations between explanatory study variables

	Gender	Age	Income	Education	Cultural activity at work
Gender (=2)	x	0.04	−0.27	0.11	0.00
Age 2006		x	0.25	−0.17	0.02
Income (In) 2006			x	0.24	0.09
Education				x	0.23
Cultural activity 06–10					x

	Non-listening manager	Psychological demands at work	Decision latitude at work	Emotional exhaustion	Depressive symptoms
Gender (=2)	0.00	0.05	−0.01	0.15	0.16
Age	0.04	−0.03	0.04	−0.03	−0.06
Income (ln)	−0.19	0.07	0.28	−0.13	−0.13
Education	−0.13	0.17	0.40	0.05	0.03
Cultural activity 06–10	−0.15	0.00	0.22	−0.08	−0.05
Non-list. boss 06–10	x	0.25	−0.30	0.30	0.30
Demand 06–10		x	0.09	0.50	0.35
Dec lat 06–10			x	−0.12	−0.15
Emotional exhaustion				x	0.71

Only subjects who have answered all questions on all the three occasions are included. 2006–2010: accumulated scores from the three study waves. This refers to cultural activity (at work), non-listening boss, psychological demands and decision latitude at work. All correlations are statistically significant

*N* = 2,088

The two outcome variables, emotional exhaustion and depressive symptoms, resemble one another in their patterns of correlations with the other study variables. Female gender, low income, low decision latitude and high level of education show significant small to moderate correlations with the outcome variables (0.03–0.16). Non-listening boss is more strongly correlated with the outcomes (0.30 for both). High psychological demands at work has the strongest correlation with the outcome variables (0.50 for emotional exhaustion and 0.35 for depressive symptoms).

Table 3 shows standardised relative regression (beta) coefficients for the associations between cultural activity and emotional exhaustion and depressive symptom scores, respectively, in the three successive stages of adjustments in cross-sectional analyses separately for the three study years. These analyses show that cultural activities at work had a more pronounced association with emotional exhaustion than with depressive symptoms and that this association was stronger in 2008 than in 2006 and 2010. Part of the effect of cultural activity on emotional exhaustion and depressive symptoms could be explained by covariation with leadership and psychosocial work environment since the magnitude of the associations decreased successively when at first “non-listening manager” and subsequently the two psychosocial work environment variables “psychological demands” and “decision latitude” were added. There was, however, a significant independent protective statistically significant association between cultural activity and emotional exhaustion even after adjustments for leadership and work environment in 2008. This was the year with the lowest unemployment and the highest number of cultural activities in work places. In 2006 and 2010 there was no statistically significant effect remaining after all adjustments (borderline significant for 2006).

**Table 3:** Cross-sectional multiple standardised relative linear regression coefficients (beta) for independent statistical “protective contribution” of cultural activities in relation to ill health in the different steps

Year	2006	2008	2010
Alternative 1. (adjusted for age, gender and income only)			
Exhaust	0.063*** (*n* = 4,955) *t* = 4.44	0.073*** (*n* = 9,381) *t* = 7.26	0.065*** (*n* = 8,671) *t* = 6.09
Depr	0.031* (*n* = 4,946) *t* = 2.28	0.051*** (*n* = 9,414) *t* = 4.96	0.042*** (*n* = 8,729) *t* = 3.98
Alternative 2. (adjusted for same as 1. plus “does your boss listen?”)			
Exhaust	0.031* (*n* = 4,826) *t* = 2.20	0.048*** (*n* = 8,564) *t* = 4.53	0.030*** (*n* = 7,964) *t* = 2.73
Depr	0.007 NS (*n* = 4,816) *t* = 0.47	0.021* (*n* = 8,586) *t* = 1.96	0.014 NS (*n* = 8,020) *t* = 1.27
Alternative 3. (adjusted for same as 2. plus demands and decision latitude at work) (*n* = 3,420)			
Exhaust	0.023(*) (*n* = 4,660) *t* = 1.70	0.029** (*n* = 8,297) *t* = 3.07	0.010 NS (*n* = 7,677) *t* = 0.97
Depr	0.006 NS (*n* = 4,655) *t* = 0.42	0.004 NS (*n* = 8,318) *t* = 0.30	0.000 NS (*n* = 7,721) *t* = 0.05

Each year has been analysed separately

(*) *p* < 0.10; * *p* < 0.05; ** *p* < 0.01; *** *p* < 0.001

The relative regression (beta) coefficient 0.073 in the first step in 2008 (alternative 1.) means that an increase of one standard deviation on the “culture at work scale” statistically corresponds to a decrease on the emotional exhaustion scale of 0.073 standard deviations. In the third step (alternative 3.) the coefficient 0.029 means that the same move on the “culture at work” scale corresponds to a decrease in emotional exhaustion of 0.029 standard deviations. Thus, the introduction of the work-related variables in this case reduces the statistical health promotion effect of cultural activity by approximately 60 %.

The prospective analyses showed that cultural activity at work in 2008 was a significant predictor of emotional exhaustion in 2010 after adjustment for emotional exhaustion in 2008 as well as age, gender, income, non-listening manager, psychological demands and decision latitude in 2008. In the corresponding analysis of the statistical power of cultural activity at work in 2006 for predicting emotional exhaustion in 2008 as well as 4 years later (2006–2010), the results were far from significant. Similarly, cultural activities at work did not predict depressive symptoms neither from 2006 to 2008 nor from 2008 to 2010. Results of the predictive analysis of emotional exhaustion from 2008 to 2010 are presented in Table 4. The independent relative beta coefficient for cultural activity is 0.021 (compared to 0.029 in the cross-sectional analysis in 2008) and statistically significant (*p* = 0.036). The strongest predictors apart from gender and age are emotional exhaustion as well as psychological demands and decision latitude at work in 2008.

**Table 4:** Multiple linear regression results for the prediction of emotional exhaustion score in 2010 from the situation in 2008

Variables	*B*	SEM *B*	*t*	*p*	Beta
Intercept	7.63	1.12	6.83	0.0001	
Gender	0.42	0.12	3.53	0.0004	0.037
Age	−0.05	0.01	9.10	0.0001	0.101
Nlog (income SEK/year)	−0.26	0.15	1.70	0.090	0.023
Non-listening manager	0.13	0.08	1.65	0.099	0.017
Psychol. demands	0.14	0.02	5.63	0.0001	0.063
Decision latitude	−0.06	0.02	2.41	0.016	0.026
Emotional exh. 2008	0.57	0.01	52.21	0.0001	0.602
Cultural activity/w	0.18	0.09	2.09	0.036	0.021

Regression coefficients (*B*) with standard errors of means (SEM), *t* value, *p* and relative beta coefficient *n* = 6,214

## Discussion

Our results show a significant cross-sectional linear relationship between cultural activities at work and mental employee health (the more frequent cultural activities the better mental health). This relationship may be stronger during periods of low unemployment than otherwise. Some of the effect may be due to the fact that cultural activities at work may be part of a good work environment with good leadership, reasonable psychological demands and good decision latitude. Expressed in another way, it could be that cultural activities at work have a beneficial effect on leadership and work environment and that this effect partly explains the association between cultural activities at work and emotional exhaustion. Research findings pointing in this direction were made by Romanowska et al. (2011). There was, however, also an independent beneficial statistical effect on emotional exhaustion of cultural activities at work for employees in the present study, at least during the good year 2008.

This study has been based upon a representative sample of working Swedish men and women. The response rate is similar to other contemporary population surveys of this kind—in the order of 60 %. In addition, there is—as in all longitudinal studies—an additional loss in the follow-up analyses. This means that we cannot claim that the study samples are fully representative of the Swedish working population, but comparable to those reported in other studies. New subjects were added in 2008 and this means that the numbers are larger in 2008 and 2010 than in 2006. Accordingly, the statistical power is lower in 2006 and in the follow-up analyses 2006–2008 and 2006–2010 than in the later analyses. However, there are large numbers in all analyses and this factor is therefore not likely to be of major importance to the interpretation. For instance, the finding that cultural activity at work had its maximum in 2008 is evident both in longitudinal and cross-sectional analyses.

The question regarding cultural activities at work is wide and in future studies more specified questions regarding kinds of cultural activities should be used.

The assessment of emotional exhaustion, depressive symptoms, psychological demands and decision latitude was performed according to accepted standardised methods. The assessment of “non-listening manager” is less established, but was made by means of a question that has been used previously in our research and has proved to be of predictive value (Oxenstierna et al. 2011).

An important message from previous research is that cultural activities must be established as repeated regular life habits. In the studies performed by Bygren et al. (1996, 2009b), attendance in cultural activities once a week during long periods is the “dosage” required for a clear long-term effect on mortality and morbidity. In the present study, most of the cultural activities at work have had a much lower frequency. The vast majority of work places reportedly organised cultural activities sometimes per year—if at all. Although according to our results even such a low frequency of activity may have some effect resulting in decreased prevalence of emotional exhaustion, it is clearly a low-frequency level. In addition, some of the activities that have been organised may not necessarily have had any health promotion effect at all. In one of our previous studies we even observed a “jealousy” effect when some employees (but not all) could take part in cultural activities. This could mean that cultural activities that are poorly organised may even have adverse health effects on employees.

The prospective analyses in which data from 2006 were used as predictors of health outcome in 2008 and similarly for data from 2008 as predictors of health outcome showed that a high level of cultural activities at work in 2008 was related significantly to a low level of emotional exhaustion score 2 years later. No such corresponding finding was made for the period 2006–2008. That cultural activities decreased during a period of unemployment and that any statistically significant cross-sectional protective effect of cultural activities could not be observed during this period could be interpreted as evidence that such activities may be particularly important during such periods. Quite to the contrary, what happened in Sweden during the economic downturn during the study period was that they decreased.
